# Method of detection, determinants and subsequent treatments for breast, cervical and prostate cancers in Edo–Benin, Nigeria

**DOI:** 10.1007/s10552-025-02001-7

**Published:** 2025-04-23

**Authors:** Gregrey Agbonvihele Oko-oboh, Anssi Auvinen, Darlington Ewaen Obaseki, Janne Pitkäniemi

**Affiliations:** 1 Health Sciences Unit, Faculty of Social Sciences, Tampere University, Arvo Ylpön katu 34, 33520 Tampere, Finland; 2 Prostate Cancer Research Center, Tampere University, Tampere, Finland; 3 Fi-Can Mid Regional Cancer Center, Tampere University, Tampere, Finland; 4 Histopathology Department, University of Benin, Benin City, Nigeria; 5 Finnish Cancer Registry, Helsinki, Finland; 6 Department of Public Health, University of Helsinki, Helsinki, Finland

**Keywords:** Cancer, Method of detection, Health check-up, Screening, Nigeria

## Abstract

**Purpose:**

This study sought to describe the methods of detection (MOD), their determinants and association with type of treatments received for breast, cervical and prostate cancers using a population-based cancer registry in Nigeria.

**Methods:**

The study analyzed incident breast (*n* = 205), cervical (*n* = 147), and prostate (*n* = 250) cancers from the Edo–Benin Cancer Registry (EBCR) from 2016 to 2018. The MOD was assigned as health check-up detected or clinically detected. Case proportion ratios (CPR) were used to compare MOD across determinant levels. Statistical association between demographic determinants and MOD were assessed using binomial regression.

**Results:**

Among the cancers recorded by the EBCR, 46% of breast (*n* = 205), 43% of cervical (*n* = 146), and 50% of prostate (*n* = 250) cases were identified through health check-ups. MOD was not significantly linked to age or marital status. Health check-ups were less common in those with less than tertiary education [breast, CPR 0.61 (95% CI 0.46–0.80), cervical, CPR 0.73 (95% CI 0.49–1.08), prostate, CPR 0.64 (95% CI 0.50–0.82)]. Significantly, more cancers detected via health check-ups were assigned to palliative care compared to clinical detection (breast: 76% vs. 58%, cervical: 80% vs. 59%, prostate: 83% vs. 64%).

**Conclusion:**

Health check-up campaigns are a key source of new cases in EBCR, but cancers detected through them are more often assigned to palliative care than those detected clinically. The findings suggest that efforts at early detection are not expressed in treatments assignment. Pre-symptomatic individuals should be encouraged to participate in health check-ups and proper treatment made available to improve these programmes.

## Introduction

Improved risk reduction methods and early diagnosis have the potential to prevent 30–50% of cancer-related deaths in low and middle-income countries, LMICs [1]. In 2020, Sub-Saharan Africa (SSA) countries reported 801,392 new cancer cases and 520,158 cancer deaths [2]. The region has the lowest availability of cancer diagnosis and treatment facilities globally [3] with a considerable number of cancers detected at advanced stages or never receive a diagnosis [4]. A 2020 evaluation of 34 health-care centers in SSA offering cancer treatment services found that 33 of these centers conducted basic histology on suspected cancer samples, and only 24 perform immunohistochemistry [1].

Detecting at early, treatable stages is crucial for cancer management. Despite the Nigerian National Strategic Cancer Control Plan (NSCCP) aiming to screen over 50% of eligible populations for cancer by 2027, the absence of a population-based screening program is noticeable [5]. Existing programmes aimed at early detection are opportunistic, widely dispersed, and mainly conducted as part of health check-up campaigns or outreach efforts [6]. These health check-up campaigns typically include medical checks for cervical, prostate, and breast cancers using the pap smear test, prostate-specific antigen test, and mammography, respectively [7]. These health check-up campaigns are not integrated into Nigeria's primary healthcare delivery system, resulting in poor utilization, quality of documentation and surveillance [6]. Previous studies in Nigeria indicate low rates of uptake of cervical cancer (range 3–39%) [8, 9], mammography for breast (range 8–22%) [10, 11], and prostate-specific antigen for prostate cancer (5%) during the health check-up campaigns [12].

Additionally, there exists a significant gap in studies examining the pathways through which cancer cases are diagnosed and registered in Nigeria. Also, the determinants influencing diagnostic pathways of cancer patients remain poorly understood. It is essential to gain a deeper insight into the determinants contributing to the variations in early cancer detection rates among different subpopulations.

This study investigated the methods of cancer detection, analysed their determinants and association with type of treatments received for breast, cervical and prostate cancers using data from the Edo–Benin Cancer Registry in Nigeria.

## Materials and methods

### Study population and data source

**Fig. 1:**
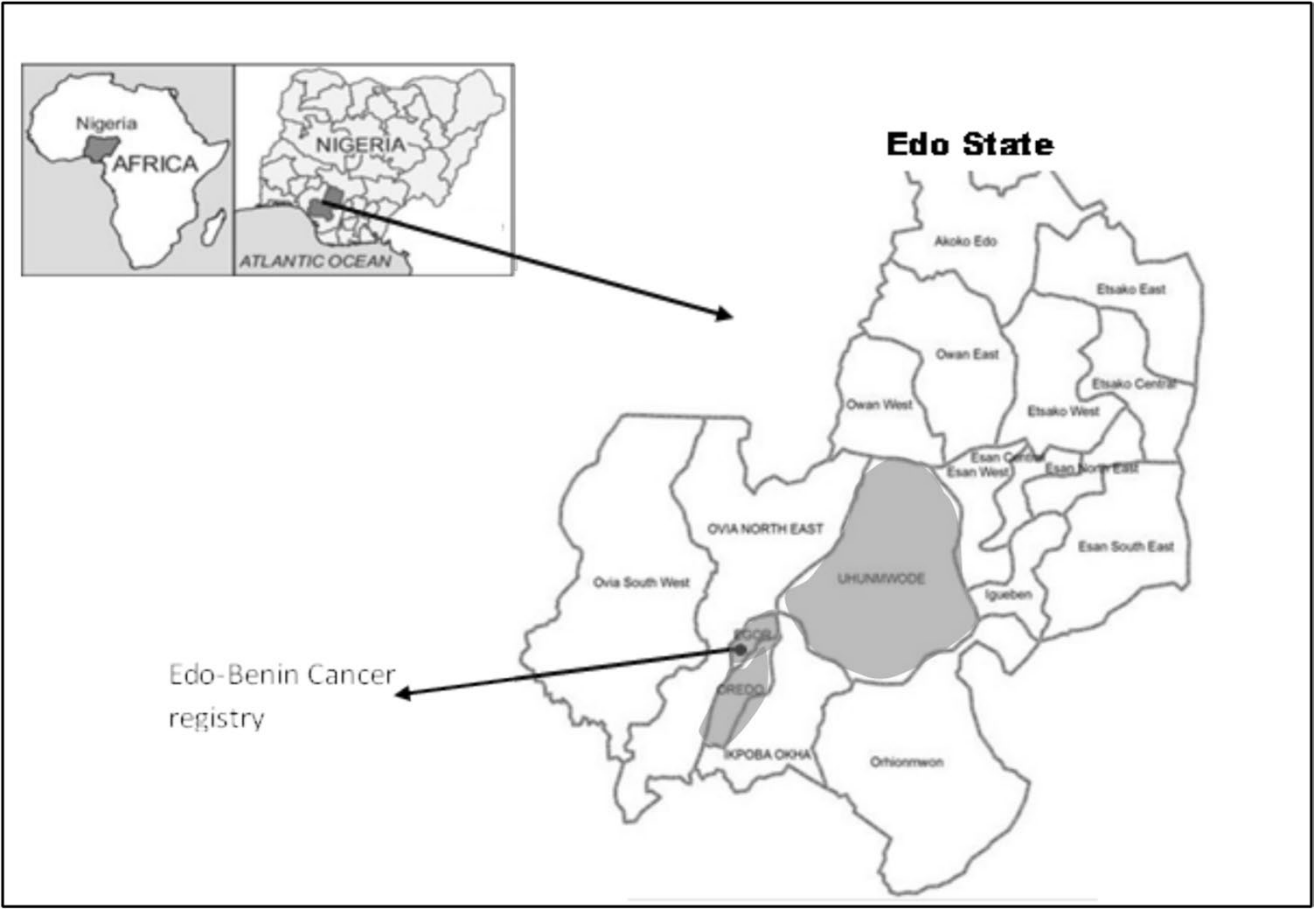
Map of Edo State, its 18 local government areas, and Edo–Benin Cancer Registry

The data for this study was from the Edo–Benin Cancer Registry, EBCR [13]. The EBCR was founded in 2008 as a hospital-based cancer registry (HBCR). In 2015, it was designated as a population-based cancer registry (PBCR) and currently, it is one of the 13 PBCRs in Nigeria. The EBCR is located at the University of Benin Teaching Hospital (UBTH). The UBTH is the only facility providing specialized cancer care services in Edo State, Nigeria [14]. It serves as a major referral center for suspected cancer cases from other health facilities, both within and outside Edo State and it can refer patients to other oncological centre for diagnostic, treatments, or rehabilitation purposes. Edo State is in the South–South geopolitical zone of Nigeria, with its capital at Benin City. It has an estimated population of 3,218,332 (1,640,461 males and 1,577,871 females) across 18 local government areas, LGAs [15]. The state accounts for 2.3% of Nigeria’s population (Fig. 1).

In this study, “health check-up” campaigns refer to early detection efforts aimed at providing community-based medical evaluations especially in underserved populations in Nigeria. Though not systematic, not nationally coordinated and targets a small proportion of the Nigerian population at a time, it is a common health programme. Health check-ups are mainly organized by non-governmental organizations, foundations, government agencies and individuals. In 2008, an early detection programme was established at the University of Benin Teaching Hospital (UBTH), Edo State. Till date, it has attended to over 8,000 participants [14]. The programme targeted adults aged 20 years and above. Risk factors information was evaluated among participants at the visit to UBTH. These include fasting serum glucose, fasting lipids profiles (total cholesterol, HDL-cholesterol, LDL-cholesterol, and triglycerides), prostate-specific antigen (men ≥ 40 years), pap smear (women 20–65 years), as well as breast examinations (performed by attending physician) performed. Positive cases were referred to the pathologist in the UBTH for case confirmation and treatment. In addition, “clinical detection” refers to cancers that are detected when patients present with symptoms in a clinical setting.

We used data on incident cancers and socio-demographic factors from 2016 to 2018 in three LGAs (Egor, Oredo, and Uhunmwonde) to ensure the dataset was reliable, and regionally representative, without being influenced by unaccounted mobility patterns or referrals from outside Edo State. Part of the registration process include self-reported address at time of cancer diagnosis enabling us to exclude cases from outside Edo–Benin. We excluded also cases from LGAs within Edo–Benin with poor regional coverage. This approach is detailed in our previous publication, “Improving Cancer Incidence Evaluation Through Local Government Area Matching: A Study of the Edo–Benin Cancer Registry in Nigeria” [16].

### Case finding

Cancer data in the EBCR are collected by trained cancer registrars from designated facilities (hospitals, medical clinics, pathology laboratories, and death certificates) according to the International Agency for Research on Cancer (IARC) guidelines [17]. Patient information for each cancer, including demographics, as well as clinical details and cancer-specific information such as topography, morphology, histological, grade, basis of diagnosis, and treatment. The data are entered into the CanReg5 software [18], following IARC guidelines, and coded using ICD-O-3, later converted to ICD-10 for statistical analysis. Conflicting information is resolved through pathologist reviews and clinical consensus [16].

### Statistical analysis

The primary outcome was the method of detecting incident cancer. The method of detection (MOD) is a routinely collected information in the EBCR (categorized and coded as; 1 = symptomatic, 2 = asymptomatic via case-finding, 3 = screening/health check-up programmes). For statistical analysis in this study, we recoded the MOD as: 0 = clinics (symptomatic), and 1 = health check-ups (asymptomatic via case-finding and screening/health check-up programmes). The socio-demographic factors considered in the analysis were sex, age, local government area (LGA), level of education and marital status. For the analysis, age was categorized as 44 years or younger, 45 to 54, 55 to 64, 65 to 74, 75 years or older; education was classified as ‘less than tertiary’ and ‘tertiary’ education; and marital status was classified into married and not married. The numbers and percentages of clinic- and health check-up-detected incident cancers are reported, and their differences by each determinant were compared using a binomial test, specifically a likelihood ratio test for homogeneity between the method of detection and the determinants. To assess disparities in detection patterns by each determinant (education level, marital status and LGAs), we estimated the case proportion ratio (CPR) adjusting for age using the log-binomial regression (a generalized linear model for binary response with log-link function). CPR is the ratios of the proportion of health check-up detected cancers by each determinant level and health check-up detected proportion at reference determinant level. The log-binomial regression model estimates adjusted prevalence ratios rather than odds ratios. This was done because the outcome (attendance of health check-up) prevalence is more than 10%. Thus, we obtain unbiased estimates of the ratios of the case proportions, making it more appropriate for interpreting relative risks in this context. We estimated also case ratio (CR) which is the proportion of those with a particular treatment conditional on cancers detected via health check-ups divided by the proportion of those with the same treatment detected via clinic. The 95% confidence intervals for the CPR and CR were estimated using logbin-package in the R software version 4.3.2. Statistical significance for all analyses was set at the 5% level.

### Ethical approval statement

This study received ethical approval from the Health Research Ethics Committee of the University of Benin Teaching Hospital (Ethical Clearance Number ADM/E 22/A/VOL.V11/148301102) on April 17th, 2023. All patient information was de-identified and patient consent was not required. Patient data will not be shared with third parties.

## Results

### Breast cancer

Of the women with breast cancer, 35% (71/205) were younger than 45 years and 46% (94/205) were detected thorough health check-ups (Table 1). Of these women, 24% had tertiary education, 95% (194/205) were married and most resided in Oredo (44%) and Egor (42%) LGAs. There were no statistically significant associations between the MOD and age at diagnosis, marital status, or place of residence. Among those with less than tertiary education, 39.6% of cases were detected through health check-ups compared to 65.3% among those with tertiary education with CPR of 0.61 (95% CI 0.46; 0.80). For non-married individuals, 45.5% of cases were detected through health check-ups, compared to 54.6% in married individuals, with CPR of 0.83 (95% CI 0.47–1.46). In Egor, Oredo, and Uhunmwonde, 44.2%, 51.7%, and 32.1% of cases, respectively, were detected through health check-ups, with CPRs of 1.17 (95% CI 0.86–1.59) for Oredo and 0.73 (95% CI 0.40–1.31) for Uhunmwonde, compared to Egor.

**Table 1:** Numbers (*n*), percentages (%) and case proportion ratio (CPR) of determinants by mode of detection for breast cancer

Determinant	Method of detection		Total	CPR (95% CI)^2^
	Health Check-up	Clinics		
	*n* (%)	*n* (%)	*n* (%)	
Age group (years)				
≤ 44	28 (39.4)	43 (60.6)	71 (34.6)	1.0
45–54	33 (54.1)	28 (45.9)	61 (29.8)	1.37 (0.95; 1.98)
55–64	16 (40.0)	24 (60.0)	40 (19.5)	1.01 (0.63; 1.63)
65–74	13 (54.1)	11 (45.8)	24 (11.7)	1.37 (0.86; 2.19)
75+	4 (44.4)	5 (55.5)	9 (4.4)	1.13 (0.51; 2.47)
Any age	94 (45.9)	111 (54.1)	205 (100.0)	–
*p*-value^1^	0.40			
Level of Education				
Less than tertiary	61 (39.6)	93 (60.4)	154 (75.9)	0.61 (0.46; 0.80)
Tertiary	32 (65.3)	17 (34.7)	49 (24.1)	1.0
Any education	93 (45.9)	110 (54.1)	203 (100.0)	
*p*-value	< 0.01			
Marital Status				
Non-married	5 (45.5)	6 (54.5)	11 (5.4)	1.0
Married	106 (54.6)	88 (45.4)	194 (94.6)	0.83 (0.47; 1.46)
Any Marital Status	111 (54.1)	94 (45.9)	205 (100.0)	
*p*-value	0.55			
LGAs				
Egor	38 (44.2)	48 (55.8)	86 (42.0)	1.0
Oredo	47 (51.7)	44 (48.3)	91 (44.4)	1.17 (0.86; 1.59)
Uhunmwonde	9 (32.1)	19 (67.9)	28 (13.6)	0.73 (0.40; 1.31)
Any LGA	94 (45.9)	111 (54.1)	205 (100.0)	
*p*-value	0.17			

^1^*p*-value for likelihood ratio test for the homogeneity between method of detection and the factors

^2^The ratio of the proportion of health check-up detected cancers by each determinant level and health check-up detected proportion at reference determinant level (case proportion ratio, CPR) adjusted for age and their 95% confidence intervals

### Cervical cancer

Among women with cervical cancer, 22.6% (33/146) were aged ≤ 44 years and 43% (63/146) were detected through health check-ups (Table 2). Only 37% of cases in women with less than tertiary education was detected through health check-ups, compared to 55% of cases in those with tertiary education, with a CPR of 0.73 (95% CI 0.49–1.08, *p* = 0.14). Among married and non-married women, 44% and 33% were detected through health check-ups, respectively, with a CPR of 1.31 (95% CI 0.51–3.37). Detection proportion through health check-ups were 51.9% in Egor, 46.7% in Oredo, and 23.5% in Uhunmwonde, with corresponding CPRs of 1.17 (95% CI 0.62–1.31) for Oredo and 0.45 (95% CI 0.23–0.88) for Uhunmwonde, compared to Egor.

**Table 2:** Numbers (*n*), percentages (%) and case proportion ratio (CPR) of determinants by mode of detection for cervical cancer

Determinant	Method of detection		Total	CPR (95% CI)^2^
	Health Check-up	Clinics		
	*n* (%)	*n* (%)	*n* (%)	
Age group (years)				
≤ 44	17 (51.5)	16 (48.5)	33 (22.6)	1.0
45–54	13 (31.0)	29 (69.0)	42 (28.8)	0.60 (0.34; 1.05)
55–64	17 (40.5)	25 (59.5)	42 (28.8)	0.79 (0.48; 1.29)
65–74	12 (57.1)	9 (42.9)	21 (14.4)	1.11 (0.67; 1.82)
75+	4 (50.0)	4 (50.0)	8 (5.5)	0.97 (0.45; 2.09)
*p*-value^1^	0.24			
Level of Education				
Less than tertiary	46 (40.0)	69 (60.0)	115 (78.8)	0.73 (0.49; 1.08)
Tertiary	17 (54.8)	14 (45.2)	31 (21.2)	1.0
*p*-value	0.14			
Marital Status				
Non-married	3 (33.3)	6 (66.7)	9 (6.2)	1.0
Married	60 (43.8)	77 (56.2)	137 (93.8)	1.31 (0.51; 3.37)
*p*-value	0.53			
LGAs				
Egor	27 (51.9)	25 (48.1)	52 (35.6)	1.0
Oredo	28 (46.7)	32 (53.3)	60 (41.1)	0.90 (0.62; 1.31)
Uhunmwonde	8 (23.5)	26 (76.5)	34 (23.3)	0.45 (0.23; 0.88)
*p*-value	0.02			
Total	63 (43.2)	83 (56.8)	146 (100.0)	

^1^*p*-value for likelihood ratio test for the homogeneity between method of detection and the factors

^2^The ratio of the proportion of health check-up detected cancers by each determinant level and health check-up detected proportion at reference determinant level (case proportion ratio, CPR) adjusted for age and their 95% confidence intervals

### Prostate cancer

Among men with prostate cancer, 2.0% (5/250) were aged ≤ 44 years and 49.6% (124/250) were detected through health check-ups (Table 3). Detection proportion through health check-ups was highest (60.0%) in those aged ≤ 44 years, and lowest (38.1%) in those aged 45–54 years. Of the men with less than tertiary education 39.7% was detected through health check-ups, compared to 62.4% in men with tertiary education, with a CPR of 0.64 (95% CI 0.50–0.82). Among married and non-married men, 49.4% and 60.0% were detected through health check-ups, respectively, with a CPR of 1.21 (95% CI 0.59–2.51). Detection proportion through health check-ups were 53.8% in Egor, 58.3% in Oredo, and 18.6% in Uhunmwonde, with corresponding CPRs of 1.08 (95% CI 0.85–1.38) for Oredo and 0.35 (95% CI 0.18–0.66) for Uhunmwonde, compared to Egor.

**Table 3:** Numbers (*n*), percentages (%) and case proportion ratio (CPR) of determinants by mode of detection for prostate cancer

Determinants	Method of detection		Total	CPR (95% CI)^2^
	Health Check-up	Clinics		
	*n* (%)	*n* (%)	*n* (%)	
Age group (years)				
≤ 44	3 (60.0)	2 (40.0)	5 (2.0)	1.0
45–54	8 (38.1)	13 (61.9)	21 (8.4)	0.63 (0.26; 1.56)
55–64	29 (55.8)	23 (44.2)	52 (20.8)	0.93 (0.44; 1.98)
65–74	40 (48.8)	42 (51.2)	82 32.8)	0.81 (0.38; 1.72)
75+	44 (48.9)	46 (51.1)	90 (36.0)	0.81 (0.39; 1.72)
*p*-value^1^	0.70			
Level of Education				
Less than tertiary	56 (39.7)	85 (60.3)	141 (56.4)	0.64 (0.50; 0.82)
Tertiary	68 (62.4)	41 (37.6)	109 (43.6)	1.0
*p*-value	< 0.01			
Marital Status				
Non-married	3 (60.0)	2 (40.0)	5 (2.0)	1.0
Married	121 (49.4)	124 (50.6)	245 (98.0)	1.21 (0.59; 2.51)
*p*-value	0.64			
LGAs				
Egor	56 (53.8)	48 (46.2)	104 (41.6)	1.0
Oredo	60 (58.3)	43 (41.7)	103 (41.2)	1.08 (0.85; 1.38)
Uhunmwonde	8 (18.6)	35 (81.4)	43 (17.2)	0.35 (0.18; 0.66)
*p*-value	< 0.01			
Total	124 (49.6)	126 (50.4)	250 (100.0)	

^1^*p*-value for likelihood ratio test for the homogeneity between method of detection and the factors

^2^The ratio of the proportion of health check-up detected cancers by each determinant level and health check-up detected proportion at reference determinant level (case proportion ratio, CPR) adjusted for age and their 95% confidence intervals

### Method of detection and treatment at cancer diagnosis

Among women with breast cancer, 16.7% (15/90) of the cancers detected through health check-ups and 40.5% (45/111) detected through clinics received surgery at diagnosis, with a case proportion ratio (CR) of 0.41 (95% CI 0.25–0.69, Table 4). In women with cervical cancer, 20.0% (12/60) of the cases detected through health check-ups and 37.3% (31/80) detected through clinics underwent surgery, with a CR of 0.52 (95% CI 0.29–0.92). In men with prostate cancer, 16.8% (20/119) of the cases detected through health check-ups and 35.7% (45/126) detected through clinics received surgery, with a CR of 0.47 (95% CI 0.30–0.75). For chemotherapy, 7.8% (7/90) of breast cancer cases detected through health check-ups received chemotherapy, compared to 1.8% (2/111) detected through clinics, with a CR of 4.33 (95% CI 0.92–20.3). There were no cases of cervical or prostate cancers that received chemotherapy. For palliative care, detection through health check-ups was higher compared to detection via clinics across all cancer types. Specifically, 75.5% (68/90) of breast cancer cases were detected via health check-ups compared to 57.7% (64/111) through clinics (CR 1.31, 95% CI 1.08–1.60). Similarly, for cervical cancer, 80.0% (48/60) of cases were detected through health check-ups versus 59.0% (49/80) through clinics (CR 1.31, 95% CI 1.05–1.62). Prostate cancer also followed the same trend, with 83.2% (99/119) detected through health check-ups, compared to 64.3% (81/126) through clinics (CR 1.29, 95% CI 1.11–1.51, Table 4).

**Table 4:** Numbers (*n*), percentages (%) and case ratio (CR) of method of detection by treatment at diagnosis for breast, cervical and prostate cancers

Treatment received at diagnosis	Breast			Cervix			Prostate		
	Health Check-up	Clinics	CR (95% CI)	Health Check-up	Clinics	CR (95% CI)	Health Check-up	Clinics	CR (95% CI)
Surgery	15 (16.7)	45 (40.5)	0.41 (0.25; 0.69)	12 (20.0)	31 (37.3)	0.52 (0.29; 0.92)	20 (16.8)	45 (35.7)	0.47 (0.30; 0.75)
Chemotherapy	7 (7.8)	2 (1.8)	4.33 (0.92; 20.3)	–	–		–	–	–
Palliative Care	68 (75.5)	64 (57.7)	1.31 (1.08; 1.60)	48 (80.0)	49 (59.0)	1.31 (1.05; 1.62)	99 (83.2)	81 (64.3)	1.29 (1.11; 1.51)
Total	90 (44.7)	111 (55.3)		60 (42.8)	80 (57.1)		119 (48.6)	126 (51.4)	
*p*-value	< 0.01			< 0.01			< 0.01		

These observations were excluded from the analysis, in radiotherapy, breast and prostate have no cancers and cervix has just two cancers in health checks. In breast there were three cases reported via clinics

*p*-value is testing likelihood ratio test for the association between treatment and the method of detection

## Discussion

Among all new cancers registered by the Edo–Benin Cancer Registry, 46% of breast cancers, 43% of cervical cancers, and 50% of prostate cancers were identified through early detection efforts as part of health check-ups. Cancers in individuals with less than tertiary level of education and those with reported residence in Uhunmwonde LGA were less likely to be detected via health check-ups than clinic settings (symptomatic detection). Importantly, 75.5% (breast), 80% (cervix) and 83% (prostate) of cancers detected through health check-ups were assigned to palliative treatment. We found that the new cancers detected through health check-ups were relatively 30% more likely (1.3-fold) to be treated with palliative care than those diagnosed through clinic settings.

Information on the method of first detection is routinely collected by the Edo–Benin Cancer Registry (EBCR) following the recommendations of the International Agency for Research on Cancer, IARC [17]. The evaluation of cancer registry data would be more beneficial for policy-makers and clinicians if it included information about the circumstances under which cancer cases were initially diagnosed in the population or how they came to medical attention. However, this information is not currently integrated into the evaluation of cancer incidence estimation by the EBCR.

The high level of awareness of breast (81%) [11], cervix (88%) [19], and prostate (58%)[12] cancers in Nigeria does not reflect in a large proportion of cancers diagnosed at early stages as observed in our study. Health check initiatives are not integrated with the healthcare system in Edo–Benin [6], as these health checks are not organized population-based screening programmes. They are mainly based on an “opportunistic” approach and information about identified cancer cases, is typically managed by the organizers of these initiatives. Linking data from these programmes to the healthcare system is further delayed by the time it takes to discern between invasive cancers from in situ carcinomas, which often requires further examination from other external sources.

The study examines methods of cancer detection across various demographic factors. Our findings suggest that regardless of age, individuals have comparable likelihoods of breast cancer diagnosis through either health check-up or clinic (via symptoms), However, MOD differs with education level. Individuals with less than tertiary education were significantly less likely to be detected through health check-up compared to tertiary-educated individuals across the three cancers. Similar to our findings, other studies showed that with increasing levels of education, individuals are more likely to undergo early detection programmes in Nigeria [20–22]. In countries with opportunistic rather than organized screening, educational attainment seems to play a less well-defined role in determining participation as is the case in Edo–Benin [23]. Instead, targeted public health education campaigns focused on cancer awareness and early detection are more effective in improving participation than relying on an individual's level of educational attainment [24–26]. The MOD also varied by place of residence. Cancers in Uhunmwode were less likely to be detected through health check-up compared to those in Egor and Oredo. Besides being a suburban and largely rural local government (LGA) compared to Egor and Oredo [15], Uhunmwode has the fewest health facilities and is the farthest from the EBCR. Revising the early detection program policies to address challenges in purpose, public awareness, and equitable service distribution across health facilities in Edo–Benin is crucial. This will improve the early identification of cancers and ensure prompt treatment.

We observed variations in the treatments received at diagnosis based on the MOD for breast, cervical, and prostate cancer cases. Breast cancer patients diagnosed through health check-ups were significantly less likely to undergo surgery at diagnosis, yet more likely to receive chemotherapy alone and palliative care. Similar findings were observed in cervical and prostate cancers. Other studies from Nigeria indicated similar pattern of cancer treatment initiation [27, 28]. Observing fewer breast cancer patients undergoing surgery and none on hormonal therapy may be related to advanced stage at diagnosis. Additionally, no cases of radiation therapy were recorded, which could reflect the limited availability of radiotherapy treatment options [29], or the absence of radiation oncologists. We are inclined to believe it is the former, for example, the Edo–Benin region of Nigeria have one radiotherapy machine with frequent downtimes (between 2016 and 2018; the period these cancers were diagnosed) [30], and three radiation oncologist [31, 32]. Although, efforts are currently in place to improve cancer care in Edo–Benin and Nigeria [6, 33]. The establishment of the Nigerian Cancer Health Fund [34], six cancer treatment centres in Nigeria (one in Edo–Benin at the University of Benin Teaching Hospital), training and employment of oncologists reflects this efforts and policy. But, the shrinking clinical oncology workforce in Nigeria [35], and on-going brain-drain of health workers leaves much to be expected [36].

Regardless of the method of first detection and the cancer site, majority of cases received palliative care only, indicating a significant proportion of late-stage diagnosis [37]. A practical reason for this could be that individuals attending early detection programs are often already symptomatic, indicating these programs are being used more as diagnostic tools rather than true preventive screenings for asymptomatic individuals. This self-selection leads to late-stage diagnoses a common occurrence in SSA [38], limiting treatment options to palliative care. Additionally, early detection programs might fail due to insufficiently sensitive diagnostic tests or inadequate follow-up on abnormal results. This is especially concerning for cervical cancer, where pre-malignant lesions, such as cervical intraepithelial neoplasia (CIN), may be missed, leading to more advanced disease at diagnosis. This highlights the need to coordinate and integrate early detection programmes into the healthcare system as practiced in Southern Africa region [39, 40]. To advocate for a government-backed national guidelines that will stipulate clear protocols for organized screening schedules, target populations, and follow-up procedures, ensuring consistency and standardization across the country.

Our research builds upon previous findings outlined in our earlier paper [16], with a focus on ensuring the coverage of registered cancers with respect to the target population. After a comprehensive literature search, we are not aware of any previous studies in Nigeria that explored the impact of early detection efforts as part of health check-up campaigns on cancer care and registration. We have collected information on the first-line treatment received, method of detection and socio-demographic factors. As a limitation, information on treatments was missing, including hormonal treatment. Beyond age and sex, our study explored the roles of education level and place of residence (local government area councils) in determining methods of cancer detection. Other socio-demographic determinants could not be explored because they were either not recorded in the Edo–Benin Cancer Registry database or when recorded, were not captured in a standardized manner (e.g., occupation) or had substantial missing data (e.g., religion), preventing meaningful analysis. Similarly, the study did not examine the role of health seeking choices and treatment decisions. Also, our study did not account for the proportion of newly diagnosed cancer patients that did not initiate treatment, which can be as much as 20% within the first 12 months in SSA, as reported by the African Breast Cancer-Disparities in Outcomes (ABC-DO) study [41]. Neither did we evaluate the use of combination therapies, nor treatment history after initiation as this were not the primary focus of our study.

A significant limitation of this paper is the absence of data on cancer staging in the EBCR. This omission is common to cancer registries in Sub-Saharan Africa [42], due to inadequate laboratory services for histological confirmation [43, 44]. To improve the availability of cancer stage information, we suggest the use of the essential TNM (eTNM) algorithm, developed by the International Agency for Research on Cancer (IARC) and the African Cancer Registry Network, AFCRN [45]. The eTNM is designed for use by cancer registries when either the traditional (histological) TNM stage have not been explicitly recorded in the patient’s record. Without stage data, we are unable to fully evaluate how early or advanced diseases influences the choice and effectiveness of detection methods and treatments which could limit the applicability of our findings to clinical practice.

Another limitation of the study is the lack of health check-up data for all subjects, including those who tested negative. This could provide more insights into the performance of health check-ups, and estimation of cancer detection rates. This is also important in evaluating overdiagnosis associated with early detection programmes especially for prostate cancer [46, 47]. However, the scope of our study was to evaluate method of cancer detection using a registry datasets which included confirmed cases of cancer. Additionally, selection bias may have skewed our findings if certain patient populations are more predisposed to undergo health checks. This is an aspect that warrants careful consideration and the need for cautious interpretation.

## Conclusions

Health check-up campaigns are major source of new cases in Edo–Benin Cancer Registry. However, cancers detected via health check-ups are assigned to palliative care more frequently than those detected clinically (via symptoms). The findings suggest that efforts of early detection (health check-ups) are not expressed in the assignment of treatments. Pre-symptomatic people should be encouraged to participate in health check-ups and proper treatment should be made available to improve these programmes.

## Data Availability

The datasets analysed during the current study are not publicly available because the cancer registry data are owned and managed by the University of Benin Teaching Hospital and the Edo–Benin Cancer Registry, which have policies limiting public access. But they are available from the corresponding author on reasonable request. Not applicable.
